# Advances in layer manganese dioxide for energy conversion and storage: mechanisms, strategies and prospects

**DOI:** 10.1039/d5sc00932d

**Published:** 2025-04-17

**Authors:** Ya-Di Zhang, Hongkun Xu, Manal S. Ebaid, Xin-Jie Zhang, Kaixin Jiang, Xuehua Zhang, Zhanhu Guo, Ben Bin Xu

**Affiliations:** a College of Petrochemical Engineering, Lanzhou Petrochemical University of Vocational Technology Lanzhou 730060 China yadizhang889@126.com; b Department of Chemistry, College of Science, Northern Border University Arar Saudi Arabia; c Department of Mechanical and Construction Engineering, Northumbria University Newcastle Upon Tyne NE1 8ST UK zhanhu.guo@northumbria.ac.uk ben.xu@northumbria.ac.uk; d Department of Chemical and Materials Engineering, University of Alberta T6G 1H9 Edmonton Alberta Canada

## Abstract

Layer manganese dioxide with its special structure, low price and large theoretical specific capacitance/capacity is considered a competitive candidate for various energy conversion and storage devices, such as supercapacitors and batteries (Li-ion, Na-ion, and Zn-ion) However, challenges such as low electronic/ionic conductivity, sluggish diffusion kinetics, and structural collapse during cycling are still the main factors limiting its practical application. A solid understanding of the correlation between structure and performance will greatly promote the performance and the further application of layer manganese dioxide. In this review, the energy storage mechanism of layer manganese dioxide in different energy storage devices is discussed in detail. Additionally, considering the current difficulties and challenges, recent advances in strategies for electrochemical performance improvement are systematically summarized, including synthetic methods, structure design, and interlayer engineering. Finally, suggestions for the future directions and developments in preparing layer manganese dioxide cathodes with high electrochemical performance are put forward.

## Abstract 2

This review summarized the energy storage mechanism, challenges and corresponding improvement strategies of layered manganese dioxide and briefly put forward research directions to enhance the electrochemical performance.

## Graphical abstract

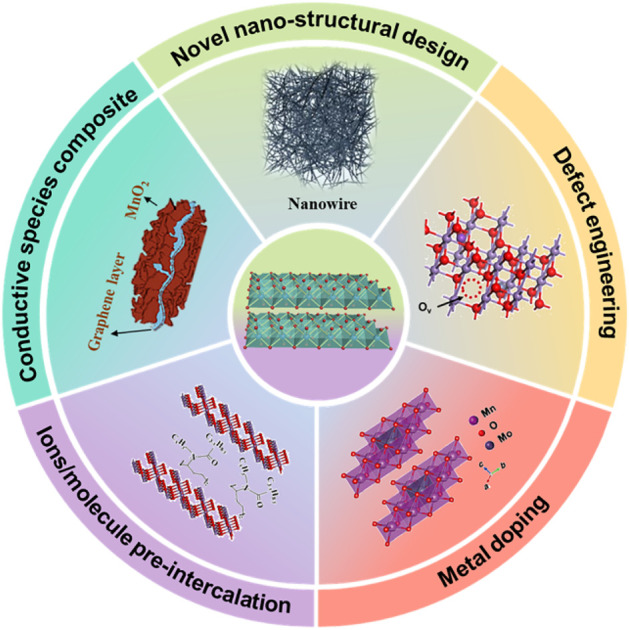

## 1. Introduction

**Fig. 1:**
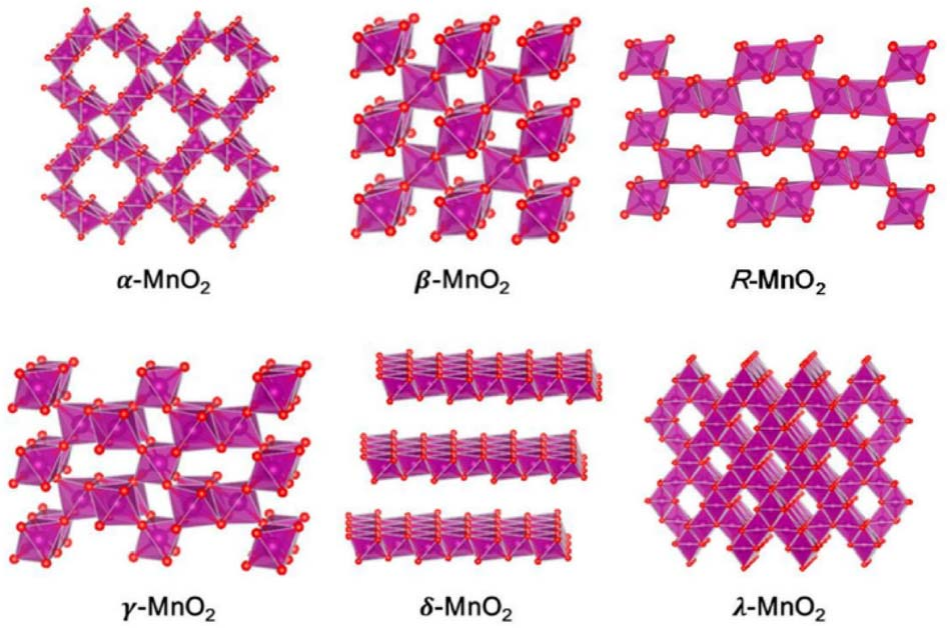
Crystal structures of MnO_2_ polymorphs (Mn: magenta and O: red). Water molecules and guest cations are omitted for clarity.^10^ Reproduced with permission from ref. 10. Copyright 2019, Sage.

With the development of renewable energy as an alternative to fossil fuels, the demand for high-efficiency energy storage/conversion devices is increasing dramatically in order to improve the efficiency of hydroelectricity, achieve the load levelling of electric power grids and improve the reliability of renewable energy, and so on.^1,2^ Until now, supercapacitors and rechargeable batteries have been considered the most representative candidates for energy conversion/storage, which can achieve reversible conversion of electrical and chemical energies.^3^ In terms of the cost, safety and environmental friendliness, MnO_2_-based materials have attracted intensive attention as electrode materials in supercapacitors and different kinds of batteries, such as Li-ion, Na-ion, Mg-ion and Zn-ion batteries. In addition, MnO_2_ has high theoretical pseudocapacitance (≈1370 F g^−1^) and a wide positive potential window (1.0 V) compared with most of the other transition metal oxides (*e.g*., NiO and Co_3_O_4_ are 0.5 and 0.45 V, respectively), thereby offering high energy density.^4–9^ However, based on various arrangements of the basic MnO_6_ octahedra units, there are six polymorphs of MnO_2_, namely, *α*-MnO_2_, *β*-MnO_2_, R-MnO_2_, *γ*-MnO_2_, *δ*-MnO_2_, and *λ*-MnO_2_,^10^ as shown in Fig. 1. Among these polymorphs, *α*-MnO_2_ is in a 2 × 2 tunnel or hollandite structure with a tunnel size of ≈0.46 nm and *δ*-MnO_2_ is in a two-dimensional (2D) layered or birnessite structure with a large interlayer separation of ≈0.7 nm, which are suitable for intercalation/deintercalation of the majority charge carrier. The narrow tunnels in *λ*-MnO_2_ and *β*-MnO_2_ give rise to a lower performance and phase transition during the initial discharge process. The structure of *γ*-MnO_2_ consists of an intergrowth between 1 × 1 and 2 × 1 tunnels that can facilitate proton intercalation in alkaline batteries.^11–14^ Obviously, MnO_2_ with different crystallographic polymorphs exhibit completely disparate redox reaction kinetics when they are used as electrode materials.

**Fig. 2:**
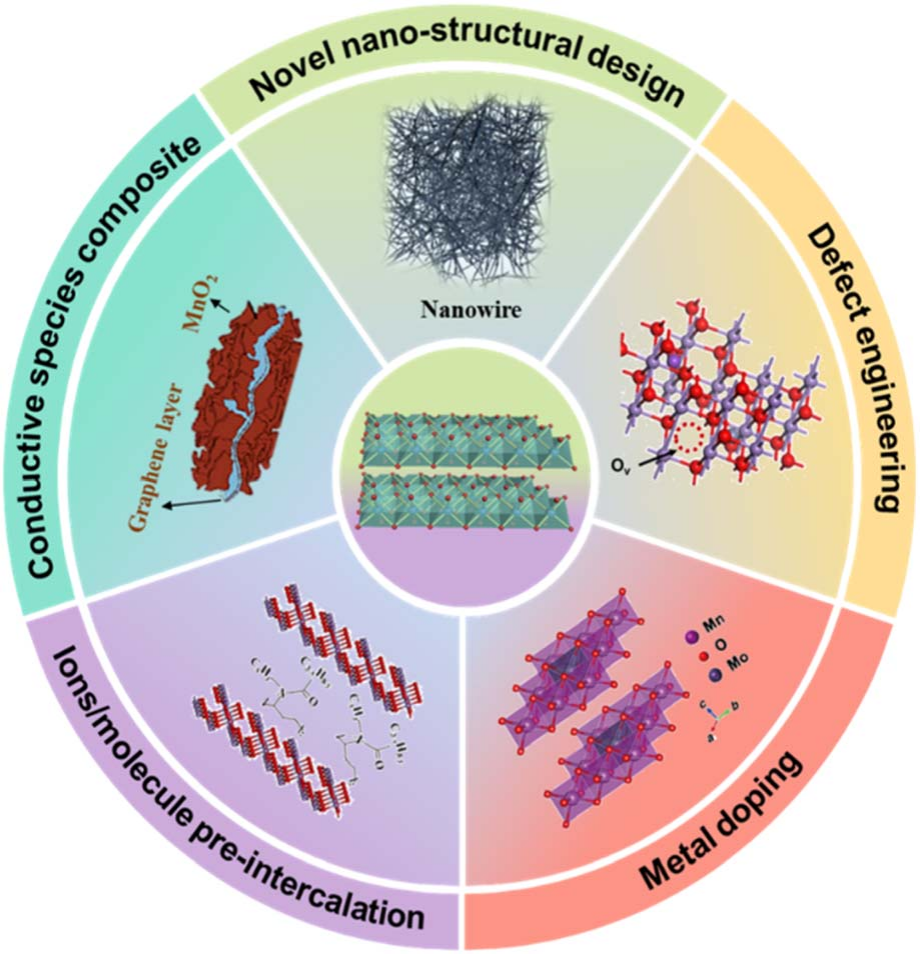
Some strategies applied on layer manganese dioxide electrode materials to improve their electrochemical performance.

Among them, layer manganese dioxide, also known as birnessite-type MnO_2_ or *δ*-MnO_2_, is the most widely used electrode material because of its adjustable interlayer spacing that could provide reasonable transport channels for insertion of alkaline ions (from monovalent to trivalent, *e.g.*, Li^+^, Na^+^, K^+^, Zn^2+^, Mg^2+^, Ca^2+^, and Al^3+^) and abound chemically active sites for redox reaction. It is widely acknowledged that layer manganese dioxide (usually represented as A_*x*_MnO_2_·*y*H_2_O (A is Na, K, or Ca)) is not a simple manganese oxide but a hydrous manganese oxide constructed with a layer of edge-sharing MnO_6_ octahedra, with various combinations of cationic species and H_2_O molecules depending upon the environment of formation.^15–18^ Even though it has been used as an electrode material for a long time, some intrinsic issues still limit its electrochemical performance, such as low electronic/ionic conductivity, structural instability upon cycling resulting from Jahn–Teller distortion and sluggish diffusion kinetics. Besides, the valence of Mn is based on the combination of Mn^2+^, Mn^3+^ and Mn^4+^, which raises the possibility of redox reactions involving ion exchange between MnO_*x*_ and electrolyte. Initially, some strategies have been introduced to improve electrochemical performance on the macro level. For example, the preparation of a carbon-containing composite,^19,20^ and the design of a nanostructure from 0D to 3D, reducing the dead mass of layer manganese dioxide.^21–23^ These pathways can increase electronic conductivity, enhance electrochemically active sites and promote material utilization, achieving 15–25% of theoretical capacitance. To further improve electrochemical performance, many studies focus on the crystal structure level, such as interlayer pre-intercalation, element doping, defect engineering, and so on, which can effectively improve the intrinsic electronic/ionic conductivity, expand the interlayer spacing and stimulate reaction kinetics. The major strategies used on layer manganese dioxide electrode materials are illustrated in Fig. 2. Nevertheless, there is still room for further improvement in electrochemical performance of layer manganese dioxide through other strategies. Herein, we reveal the intrinsic mechanisms of layer manganese dioxide in various energy conversion and storage devices and summarize the previously reported solutions. Furthermore, bottlenecks in electrochemical performance of layer manganese dioxide electrode materials and the corresponding solutions are proposed to guide future research.

## 2. Energy storage mechanism of layer manganese dioxide

### 2.1 Surface absorption and desorption mechanism

Two effects typically occur when the electrolyte ions are in contact with the electrode interface: one is the adsorption behavior of fully solvated ions on the electrode interface through electrostatic interaction, resulting in a non-Faraday electric double layer (EDL) capacitance, and the other is the specific adsorption of desolvated or partially solvated ions on the electrode interface through chemical bonding, resulting in a Faraday pseudocapacitance. In fact, EDL capacitance exists in nearly all electrodes, similar to a parallel-plate capacitor. In some studies, an almost rectangular profile was delivered in cyclic voltammetry (CV) curves of layer manganese dioxide in neutral electrolyte, analogous to non-faradaic energy storage mechanisms.^24^ Researchers have investigated whether EDL capacitance is proportional to the specific surface area available for the active material,^25–27^ according to *C* = *Aε*_r_*ε*_0_/*d*, where *A* is the specific surface area of the electrode accessible to the electrolyte ions, *ε*_r_ is the electrolyte dielectric constant, *ε*_0_ is the permittivity of a vacuum, and *d* is the effective thickness of the EDL. The specific surface area is a factor that must be considered in the process of material preparation.

### 2.2 Surface redox reaction mechanism

Studies have shown that layer manganese dioxide exhibits characteristics related to pseudocapacitance, such as changes in structure, local bonding and Mn oxidation state, which was first described in acidic electrolytes.^28,29^ Subsequently, a similar mechanism was found in mild electrolyte.^30^ This is mainly due to the adsorption of alkaline cations (Li^+^, Na^+^, K^+^, *etc.*) in the electrolyte with the opposite electrical properties of O in the MnO_6_ octahedron. Then, the charge is transferred to the adjacent Mn atom, its valence state is changed from +4 to +3, and the reaction is described below:(MnO_2_)_surface_ + C^+^ + e^−^ → (MnOOH)_surface_where *C*^+^ = Li^+^, Na^+^, and K^+^. It should be noted that the reaction only occurs on the surface atom or in a thin layer of layer manganese dioxide. Therefore, constructing an ultra-thin layer is an effective strategy to improve the capacitance. The redox reaction is very fast and it is difficult to directly distinguish from EDL capacitance, but it involves a chemical reaction resulting in higher capacitance. Additionally, the choice of potential window will cause a difference in the energy storage mechanism. For example, Xia *et al.* discussed the electrochemical behavior of the birnessite Na_0.5_MnO_2_ in different potential windows, finding that the reversible redox reaction of Mn^3+^/Mn^4+^ was accompanied by the insertion/extraction of Na^+^ in a window of 0–1.0 V, the deintercalation and intercalation of Na^+^ results in the large potential window of 0–1.3 V.^23^ As reported in the literature,^4,31,32^ when the upper cutoff potential is set below 0.8 or 1 V, Mn^3+^/Mn^4+^ redox reaction would not be not complete, and the energy storage of layer MnO_2_ would be dominated by pseudocapacitive resulting from rapid surface Faraday reactions. When the potential window is widened to 1.0–1.4 V, a hybrid energy storage mechanism with synergistic pseudocapacitance and ion intercalation/deintercalation occurs. Oxygen vacancies usually reduce the Jahn–Teller distortion by reducing the coordination symmetry of MnO_6_ octahedron and inhibiting manganese dissolution and structural collapse at high potentials. Besides, element doping can reduce the band gap of MnO_2_ and reduce polarization, resulting in the layered structure working under a wide potential window. Therefore, the influence of various factors on the energy storage mechanism should be considered comprehensively.

### 2.3 Cation intercalation/deintercalation mechanism

**Fig. 3:**
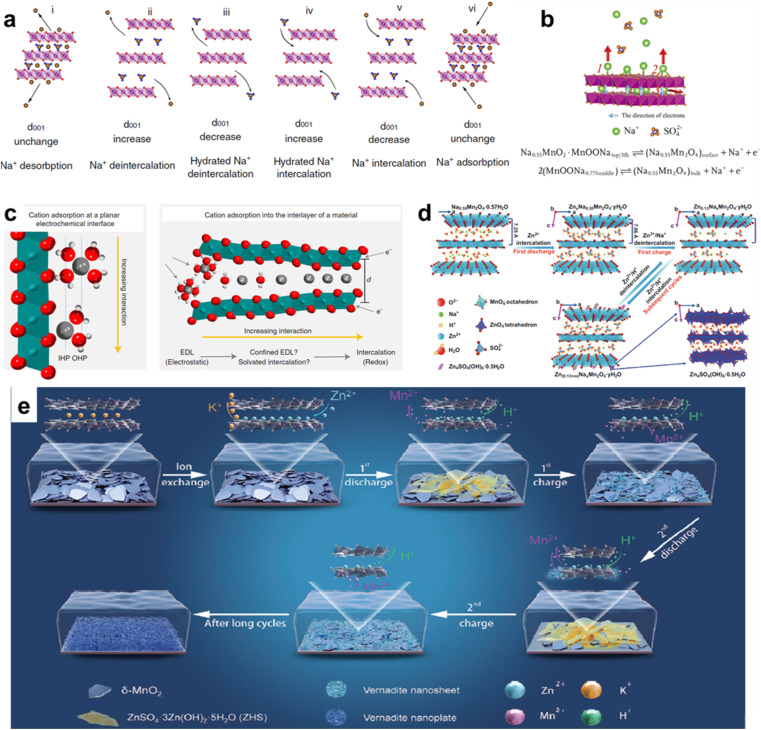
(a) Schematic of the motion of Na-ions and water during charging and discharging processes.^34^ Reproduced with permission from ref. 34. Copyright 2019, Springer Nature. (b) Movement tendency of ions and electrons during charging and discharging processes, and the whole charge storage of NMO in Na_2_SO_4_ solution.^36^ Reproduced with permission from ref. 36. Copyright 2019, Wiley-VCH. (c) Schematic of the degree of ion interaction with the electrode surface.^37^ Reproduced with permission from ref. 37. Copyright 2021, Springer Nature. (d) Schematic of the displacement/intercalation reaction mechanism in the first cycle, and the insertion/extraction mechanism of zinc ions in subsequent electrochemical discharge/charge processes.^38^ Reproduced with permission from ref. 38. Copyright 2020, Springer Nature. (e) Illustration of the reaction process of *δ*-MnO_2_ electrode in AZIBs.^44^ Reproduced with permission from ref. 44. Copyright 2024, Wiley-VCH.

In recent studies, another important energy storage mechanism in layer manganese dioxide is the (de)intercalation of alkaline cations in the bulk of the material. The reaction involved in the charge/discharge process is shown below:MnO_2_ + C^+^ + e^−^ → MnOOCwhere *C*^+^ = H^+^, Na^+^, K^+^, and Li^+^. This process is more like a battery behaviour than a capacitive behaviour, which is limited by ion diffusion within the crystalline framework of active material. Currently, a CV investigation proposed by Dunn is employed to distinguish diffusion-controlled contribution and surface capacitive contribution,^33^ as shown in the following formula:*i* = *aν*^*b*^where *i* is the current (A), *ν* is the sweep rate (V s^−1^), *a* and *b* are adjustable parameters, and *b* value has special significance. In particular, *b* = 0.5 means that the redox reaction is controlled by ion diffusion, while *b* = 1 means a typical capacitive behavior. The capacitive contribution includes EDL capacitance and pseudocapacitance. To enhance the electrochemical performance of layer manganese dioxide, pre-intercalated MnO_2_ (cation or polymer) has been widely studied by researchers, and the corresponding energy storage mechanism has been investigated. Teng *et al.* have explored the sodium storage mechanism of sodium rich disordered birnessite (Na_0.27_MnO_2_).^34^*In situ* X-ray diffraction illustrated that sodium ion and water co-deintercalated in the bulk of Na_0.27_MnO_2_ during high voltage discharge (Fig. 3a), which prevented excessive increase of layer spacing and stabilized the layer structure, benefiting in a large voltage (2.5 V). Lin *et al.* proposed a simple “hydrothermal insertion of potassium” strategy that effectively increased the potassium content in *δ*-MnO_2_.^35^*In situ* X-ray diffraction (XRD) and theoretical calculations exploring the charge storage mechanism shows that the multi-stage charging and discharging platforms appears with increasing potassium content, proving K^+^ intercalation/deintercalation behavior in the layered matrix. Moreover, the remaining K^+^ between the layers can inhibit the collapse of the layered structure in the charging state, ensuring a long cycle life. Zhang *et al.* reported the charge storage mechanisms of 2D cation intercalated manganese oxide in neutral electrolyte (NaSO_4_).^36^ It showed surface-controlled pseudocapacitive behaviour at low potential (0–0.8 V), while intercalation pseudocapacitive behaviour became dominant when the potential was higher than 0.8 V (Fig. 3b).

To elucidate the relevant capacitance mechanism in layer manganese dioxide, plenty of experimental and computational studies were carried out by the Augustyn group.^37^ It has been found that high efficiency charge storage in neutral electrolyte can be achieved through the combination of water molecules and confined alkaline cations. The weight change before and after charge and discharge measured by electrochemical quartz crystal microbalance (EQCM) showed that the storage of capacitive/charge in birnessite was controlled by interlayer cation (de)intercalation (Fig. 3c). At the same time, the distance between the intercalated cation and birnessite increased due to the presence of nanoconfined interlayer structure water, resulting in reduced interaction and exhibiting capacitive properties which mediated the interaction between the intercalated cation and the main host of birnessite and led to minimal structural changes. Zhai *et al.* successfully synthesized the layered Na_0.55_Mn_2_O_4_·0.57H_2_O (NMOH) co-intercalated with sodium ion and crystal water and explored the zinc ion storage mechanism.^38^ In this research, a displacement/intercalation mechanism was confirmed for the first time. In detail, Zn^2+^ replaced some Na^+^ and remained in the interlayer, which played a supporting role to stabilize the layered structure and promoted the subsequent Zn^2+^/H^+^ (de)intercalation process in the first electrochemical process (Fig. 3d) so that the battery obtained a high specific capacity and excellent cycle stability. In addition, Na^+^ removed from NMOH in the first cycle will be re-adsorbed to the anode surface during the discharge process, generating pseudocapacitance to increase the specific capacity of the electrode material. The same energy storage mechanism was also confirmed in the literature.^16,39–41^ A cation (de)intercalation mechanism was also illustrated in a Mg/Al-pre-intercalated MnO_2_ battery.^42,43^ However, Cui *et al.* suggested that Zn^2+^ initially irreversibly inserted to replace the interlayer K^+^ without capacity contribution, and the charge storage was mainly dominated by the electrochemical H^+^ intercalation/extraction, the electrodissolution of *δ*-MnO_2_ and the electrodissolution-electrodeposition of vernadite rather than Zn^2+^/H^+^ (de)intercalation process (Fig. 3e).^44^ These two distinct conclusions should be further probed.

## 3. Strategies to enhance electrochemical performance

Electronic conductivity, active site and specific surface area of electrode materials are closely related to electrochemical performances of energy storage/conversion devices, which mainly determine the charge transfer ability and diffusion of electrolyte ions at the electrode/electrolyte interface during the charge/discharge process. Given this, some strategies were attempted to alleviate the above situation, such as the conductive species composite, pre-intercalation, transition metal-doping, defect engineering, and so on, and these will be reviewed in detail.

### 3.1 Layer manganese dioxide/conductive species composite

Graphene, carbon nanotubes (CNTs), and porous carbon are commonly used conductive backbone or matrix compounded with layer dioxide, creating a high specific surface area abound electrochemically active sites, and high electronic conductivity.^45–48^ The formation of layer manganese dioxide/carbon composite is mainly based on *in situ* redox deposition in acid solution.^49,50^ The redox reaction of KMnO_4_ and carbon is expressed as:4KMnO_4_ + 3C + 2H_2_SO_4_ → 4MnO_2_ + 3CO_2_ + 2MnSO_4_ + 2H_2_O

**Fig. 4:**
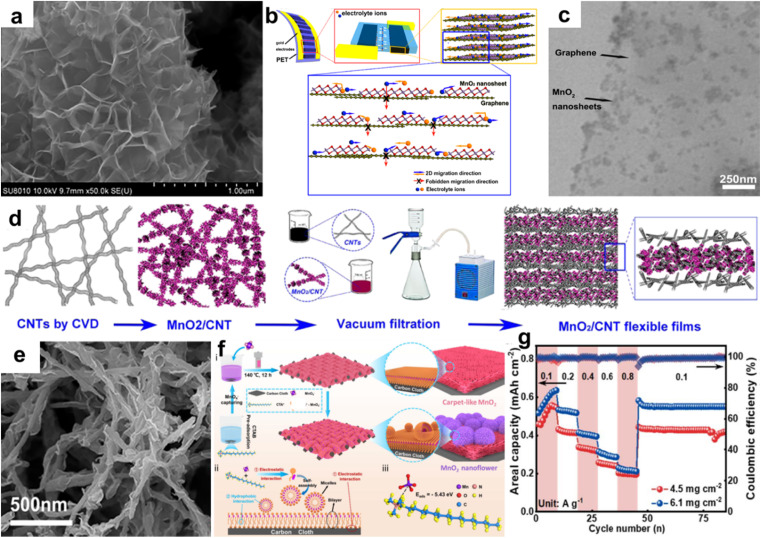
(a) SEM image of MnO_2_/graphene flower.^51^ Reproduced with permission from ref. 51. Copyright 2022, Frontiers Media Sa. (b) Schematic of the ultra-flexible planar supercapacitor. (c) TEM image of the 2D hybrid structure with *δ*-MnO_2_ nanosheets integrated on graphene surfaces.^52^ Reproduced with permission from ref. 52. Copyright 2013, the American Chemical Society. (d) Schematics of the stacked films' preparation process. (e) SEM image of MnO_2_@CNT composites.^53^ Reproduced with permission from ref. 53. Copyright 2016, the American Chemical Society. (f) Synthesis process and mechanism of MNSMO@CC. (g) Rate performance with different mass loadings.^55^ Reproduced with permission from ref. 55. Copyright 2024, the Royal Society of Chemistry.

In the process, carbon acts as a reducing agent and substrate, and electrons transfer from carbon to MnO_4_^−^, forming a thin, uniform and continuous MnO_2_ coating. It should be noted that carbon materials need to have some defects to facilitate the growth of MnO_2_. Therefore, a hydrothermal process, microwave-assisted irradiation, and/or a refluxing technique are usually carried out to synthesize layer manganese dioxide/carbon composite. Liu *et al.* reported *δ*-MnO_2_/graphene flower-like microspheres using thermally-exfoliated graphite.^51^ SEM images (Fig. 4a) showed that the resulting MnO_2_ nanosheets arrays dispersed on the graphene surface exhibited a honeycomb-like structure. The composite delivered a maximum specific surface area of 252.3 m^2^ g^−1^, resulting in excellent performances such as cycle stability and capacitance retention. Peng *et al.* prepared birnessite/graphene composite with a layer-by-layer structure to assemble planar supercapacitors (Fig. 4b).^52^ The novel hybrid nanostructure with uniform MnO_2_ distribution (Fig. 4c) introduced more electrochemically active surfaces for electrolyte ions and extra interfaces to facilitate charge transport. Additionally, a graphene film with good flexibility can adapt to various bending and deformation and is commonly used for the preparation of flexible electrochemical devices. Based on the high length–diameter ratio of CNTs, Li *et al.* synthesized MnO_2_/CNTs composite with uniformly cross-linked MnO_2_ nanoflakes anchored on CNTs through a modified one-pot reaction process.^50^ Wu *et al.* used CNTs prepared by the chemical vapor deposition (CVD) method as a conductive substrate material to support *δ*-MnO_2_.^53^ Then, flexible MnO_2_@CNTs/CNTs film was prepared by vacuum filtration with the addition of CNTs (Fig. 4d). As shown in Fig. 4e, *δ*-MnO_2_ grew uniformly on the surface of carbon nanotubes. The porous self-supported MnO_2_@CNTs/CNTs film electrode showed excellent pseudocapacitance behaviour, including high volume capacitance of about 177.5 F cm^−3^ at 0.2 A g^−1^ and good stability of 90% capacitance retention after 5000 cycles at 50 mV s^−1^. Meanwhile, there was almost no performance fading at different bending sates, illustrating a high flexibility. Porous carbon has a large specific surface area and abundant pore structure, which facilitates the flow of electrolyte ions while increasing the specific surface area. An *et al.* used carbonized conjugated microporous polymer hydrogels as conductive substrate and matrix to support manganese dioxide to obtain C-CMPAs@MnO_2_ composite as a binder-free cathode for a zinc-ion battery.^54^ The special structure increased the contact area of the electrode/electrolyte, provided abundant active sites, and effectively shortened the zinc ion insertion/extraction path. The test results showed that C-CMPAs@MnO_2_ composite exhibited a high specific capacity (670.7 mA h g^−1^ at 0.1 A g^−1^) and an excellent rate performance (149.2 mA h g^−1^ at 3 A g^−1^). To accelerate the electron migration and transfer rate of Zn^2+^/H^+^, carbon cloth (CC) was employed as a collector to support tailoring hierarchical MnO_2_ (MNSMO@CC) resulting from leveraging the nanomicellar properties of cetyltrimethylammonium bromide (CTAB) (Fig. 4f(i–iii)).^55^ The mass loading increased to 6.1 mg cm^−2^, and the areal capacitance reached 0.64 mA h cm^−2^ at 0.1 A g^−1^ with a good reversibility. Additionally, a long cycle lifespan was achieved, with a capacity retention of 81.8% at 0.8 A g^−1^ after 1300 cycles, owing to a buffer of CC against *δ*-MnO_2_ structural strains during ion intercalation. Besides carbon substrate, nickel foam,^56,57^ stainless steel,^58^ Pt,^59^ Au,^60–62^ and conductive polymer (*e.g.*, polyaniline, polypyrrole, polydopamine)^21,63–67^ are used as substrates for supporting layer manganese dioxide to improve the electrochemical performance. The preparation of layer manganese dioxide/conductive species composite mainly improves the electronic conductivity, while the introduction of carbon materials inevitably increases the quality of electrode, which is not conducive to the improvement of specific capacity, making it particularly important to modify/design the structure of layer manganese dioxide itself.

### 3.2 Novel nano-structural design

The energy storage mechanism in layer manganese dioxide involves EDL and pseudocapacitance behavior on the surface, and the interlayer intercalation–deintercalation behavior of alkaline ion. Therefore, it is an effective strategy to reduce the dead mass in the bulk of layer manganese dioxide to enhance the electrochemical performance. Nano-structural design can enlarge the electrode/electrolyte contact area and shorten the diffusion path of electrolyte ions, which will increase the utilization of Mn in the interlayer and eliminate the low intrinsic conductivity of layer manganese dioxide caused by a high content of crystal water, sparse atoms and poor crystalline degree. Layer manganese dioxide are easily fabricated with various nanostructures through different synthetic methods and processing parameters, such as 0D nanoparticles (nanospheres) and dots, 1D nanowires and nanorods, 2D nanosheets and nanobelts, and 3D nanoflowers self-assembled from the above low-dimensional nanostructured building blocks and porous nanostructures.^68–72^ In this section, we provide a detailed review on the effect of the nanostructure of layer manganese dioxide on its electrochemical properties.

**Fig. 5:**
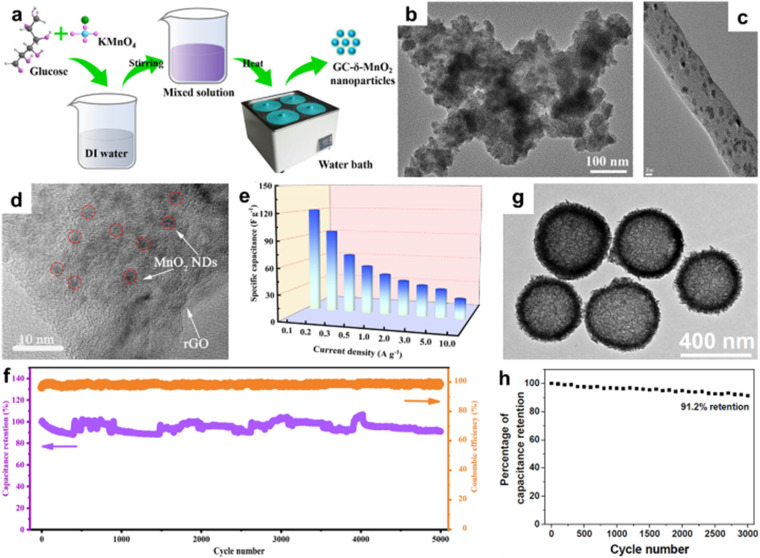
(a) Schematic illustration of the preparation of GC-*δ*-MnO_2_ nanoparticles. (b) FETEM image of GC-*δ*-MnO_2_-2.^73^ Reproduced with permission from ref. 73. Copyright 2022, Springer. (c) HR-TEM image of MnO_2_ nanoparticles/carbon nanofiber composite.^74^ Reproduced with permission from ref. 74. Copyright 2022, the American Chemical Society. (d) TEM image of MnO_2_ NDs/rGO. (e) Rate performance at varying current densities. (f) Cyclic performance and coulombic efficiency at 1.0 A g^−1^ of MnO_2_ and MnO_2_ NDs/rGO.^75^ Reproduced with permission from ref. 75. Copyright 2023, Elsevier. (g) TEM image of *δ*-MnO_2_ HMS. (h) Cyclic performance of *δ*-MnO_2_ HMS at 5 A g^−1^.^76^ Reproduced with permission from ref. 76. Copyright 2018, Elsevier.

0D nanoparticles are conducive to a good distribution on conductive matrix, which can improve the conductivity and the utilization of electrode material. Xie *et al.* reported graphite-like carbon-decorated *δ*-MnO_2_ composite by a water bath method that was applied to zinc-ion batteries (Fig. 5a).^73^ The size of *δ*-MnO_2_ nanoparticles was about 25 nm (Fig. 5b), with abundant active sites and a short ionic diffusion path, facilitating high specific capacity and excellent cyclic stability. Yun *et al.* coated MnO_2_ nanoparticles on porous carbon nanofiber (CNF) matrix (Fig. 5c).^74^ This strategy maximized the synergistic effect of the pseudocapacitance of MnO_2_ and the electric double-layer capacity induced by the highly porous surface. Tang *et al.* synthesized novel MnO_2_ nanodots/reduced graphene oxide (MnO_2_ NDs/rGO) composite *via* the hydrothermal approach followed by ultrasonic treatment (Fig. 5d).^75^ The synergistic effect of small-sized MnO_2_ nanodots and conductive rGO accelerated the diffusion kinetics and charge transfer ability of ZIBs, resulting in high rate performance (124 mA h g^−1^ at 2.0 A g^−1^) and cycling stability (90.1% capacity retention after 1000 cycles at 1.0 A g^−1^) (Fig. 5e). Xiao *et al.* prepared hierarchical mesoporous *δ*-MnO_2_ hollow microspheres (*δ*-MnO_2_ HMS) using a hydrothermal method (Fig. 5f).^76^ The hierarchical mesoporous shell (Fig. 5g) with a large specific surface area provided abundant electroactive sites and accessibility for electrolyte ions, guaranteeing a high utilization of active materials. Furthermore, interior cavities within *δ*-MnO_2_ HMS regarded as ion reservoirs can shorten the diffusion path of the electrolyte and provide sufficient ions. Based on this special feature, *δ*-MnO_2_ HMS delivered a long cycle life (only 8.8% capacitance fading after 3000 cycles at 5 A g^−1^) (Fig. 5h). It needs to be stressed that the small particle radius of nanoparticles leads to low thermodynamic stability and easy agglomeration, making them not conducive to synthesis, preparation and application.

**Fig. 6:**
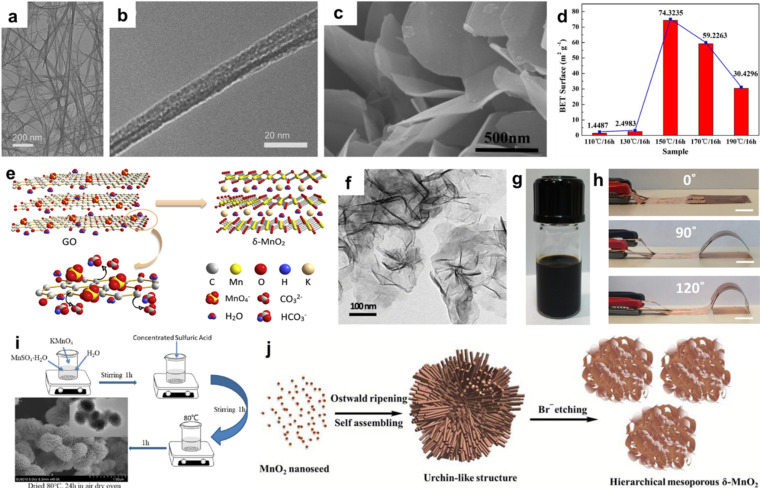
(a and b) TEM images of KMO NNT.^69^ Reproduced with permission from ref. 69. Copyright 2021, Elsevier. (c) SEM image of *δ*-MnO_2_ at 150 °C, (d) Specific surface area of *δ*-MnO_2_ at various temperatures.^87^ Reproduced with permission from ref. 87. Copyright 2016, Elsevier. (e) Illustration of the formation of *δ*-MnO_2_ nanosheets. (f) TEM image of the as-prepared *δ*-MnO_2_ nanosheets.^88^ Reproduced with permission from ref. 88. Copyright 2019, Elsevier. (g) Image of the formulated *δ*-MnO_2_ nanosheet ink. (h) Optical images of MSC bent under different angles.^89^ Reproduced with permission from ref. 89. Copyright 2018, Elsevier. (i) Schematic for the preparation of spherical *δ*-MnO_2_ nanoflowers.^90^ Reproduced with permission from ref. 90. Copyright 2024, the American Chemical Society. (j) Plausible mechanism involved in the growth of mesoporous *δ*-MnO_2_ nanostructures.^91^ Reproduced with permission from ref. 91. Copyright 2016, the Royal Society of Chemistry.

1D layer manganese dioxide such as nanowires and nanorods have high length–diameter ratios and nanoscale dimensions that can realize a short ion diffusion distance, so it is favored by researchers.^77–80^ The most common method for preparing 1D layer manganese dioxide is the hydrothermal method. Liu *et al.* employed a simple hydrothermal method to prepare a 1D-3D interconnected *δ*-MnO_2_ nanowire network (KMO NNT).^69^ KMO NNT shows 1D–3D network hybrid architecture (Fig. 6a and b), consisting of the interconnected nanowires with average estimated diameters of 10–70 nm. KMO NNT acts as cathode material for Zn-ion batteries, exhibiting a reversible capacity of 342 mA h g^−1^ at 0.162C, 150 mA h g^−1^ at 6.494C, illustrating a good rate performance, which benefited from an enhanced contact surface and short ion diffusion pathway. Like nanoparticles, 1D nanowires and nanofibers can be combined with conductive substrates to enhance the performance.^81,82^ Nanofilms and nanoflakes are the most popular morphology types due to the flexibility and the large surface areas that improve abundant redox-active sites compared with 1D and 2D layer manganese dioxide such as nanosheets.^83–86^ Xie's group employed the hydrothermal method at different temperatures to prepare *δ*-MnO_2_ with various microstructures.^87^ Porous *δ*-MnO_2_ nanosheets were obtained when the hydrothermal process was carried out at 150 °C for 16 h (Fig. 6c). The BET results showed that *δ*-MnO_2_ nanosheets delivered the largest specific surface area (Fig. 6d). Guo *et al.* reported ultrathin *δ*-MnO_2_ nanosheets with a thickness of about 2–4 nm *via in situ* reduction using graphene oxide as a reductant and self-sacrificing template (Fig. 6e and f).^88^ The electrochemical performance of Zn-ion battery was better than *δ*-MnO_2_ microspheres due to favourability of the ultrathin nanosheets for fast diffusion during cycling. In addition, a *δ*-MnO_2_ nanosheet flexible electrode was fabricated by Wang *et al.* using inkjet printing, which can be used as binder-free electrode to assemble all-solid-state symmetrical micro-supercapacitors with highly mechanical flexibility (Fig. 6g and h).^89^ The major advantage of hierarchical 3D materials is that they have a uniform pore size, high surface area and facile transport of electrolyte ions. Zhang *et al.* displayed spherical *δ*-MnO_2_ nanoflowers with a high specific surface area (228.0 m^2^ g^−1^) and abundant mesopores (primarily around 4 and 50 nm, Fig. 6i).^90^*δ*-MnO_2_ serving as an electrode material for the supercapacitor with a nanoflower structure can achieve higher bulk phase utilization and fast kinetics according to the linear potential sweep technique. Furthermore, Bag and Raj synthesized hierarchical mesoporous *δ*-MnO_2_ using the thermodynamically favourable redox reaction (Fig. 6j).^91^ The novel structure gave *δ*-MnO_2_ a high specific surface area of 238 m^2^ g^−1^ and an average pore size of 3.6 nm, which was beneficial for charge transfer/ion diffusion during charge storage. A specific capacitance of 364 F g^−1^ at a current density of 1 A g^−1^ was obtained, which could be further enhanced by reducing the size of *δ*-MnO_2_ clusters to increase the redox-active sites. Apart from the above methods, 3D *δ*-MnO_2_ microflowers can be prepared by the microwave and hydrothermal methods.^92,93^

According to the above discussions on structural designs for layer manganese dioxide nanomaterials, a perfect structure should include the following features: (1) The size of nanostructure unit (diameter of nanodots/nanofiber, thickness of nanosheet/nanofilm) should be refined to 2–4 nm as much as possible to provide accessible surface area and redox active sites; (2) a conductive matrix should be used so that the layer manganese dioxide based nanomaterials grow better, ensuring high electric conductivity; (3) reasonable pore size and distribution should be maintained, providing a smooth diffusion path for electrolyte ions.

### 3.3 Defect engineering

**Fig. 7:**
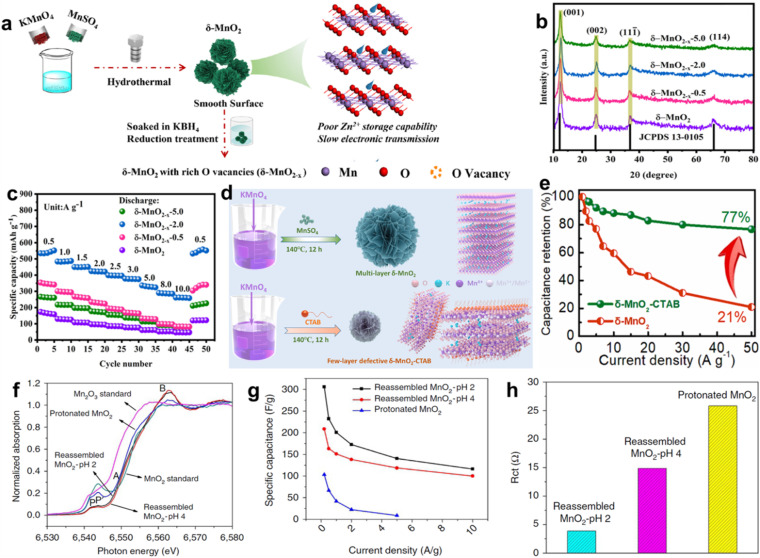
(a) Schematic of the preparation procedure for *δ*-MnO_2_ with rich oxygen vacancies (*δ*-MnO_2−*x*_). (b) XRD patterns of all samples. (c) Rate performance of *δ*-MnO_2−*x*_-2.0.^97^ Reproduced with permission from ref. 97. Copyright 2023, Springer. (d) Preparation scheme of *δ*-MnO_2_ and *δ*-MnO_2_-CTAB samples. (e) Specific capacitance values of *δ*-MnO_2_-CTAB and *δ*-MnO_2_ (different current densities).^99^ Reproduced with permission from ref. 99. Copyright 2023, Elsevier. (f) XANES spectra of protonated MnO_2_, pH = 2 and 4 treated reassembled MnO_2_. (g) Comparison of specific capacitance of the samples as a function of current density. (h) Comparison of the charge transfer resistance among the three samples.^103^ Reproduced with permission from ref. 103. Copyright 2016, Springer Nature.

The reported specific capacitance/capacity of layer manganese dioxide are much lower than the theoretical value and the rate capability is poor at high current density, which are mainly caused by intrinsic inferior electronic/ionic conductivity and sluggish reaction kinetics. Among various strategies, defect engineering including anion and cation vacancies is an outstanding approach to boost the electrochemical performance. Oxygen vacancy is the most widely explored anion vacancy in transition metal oxides because of its low energy and easy fabrication. The existence of oxygen vacancies may have a great influence on the physical and chemical properties of layer manganese dioxide, such as the change of the oxidation states of Mn ions (Mn^2+^, Mn^3+^, and Mn^4+^), the modulation of the band gap, and the variation of the charge carrier density, fundamentally improving the electrical/ionic conductivity.^94–96^ Additionally, the introduction of oxygen vacancies can produce more electrochemically active sites for the accession of electrolyte to active materials, attributed to the enhancement of capacitance/capacity. In fact, the concentration of the oxygen vacancy has a double-edged sword effect on the performance of the MnO_2_ electrode. For example, a moderate vacancy can significantly improve the electrochemical activity and conductivity, but excessive vacancy will damage the structural stability‌. Li's group fabricated flower-like *δ*-MnO_2_ nanostructures with different oxygen vacancy concentrations controlled by the reduction treatment time in KBH_4_ solution (Fig. 7a).^97^ The wider and weaker diffraction peak intensity in XRD patterns illustrated typical disordered structures owing to the introduction of vacancy defects (Fig. 7b). Although the Mn^3+^/Mn^4+^ ratio increased with higher oxygen vacancy concentrations to provide abundant active sites, Mn^4+^ balanced the introduced oxygen vacancies, resulting in structural instability. Additionally, extremely robust H^+^/Zn^2+^ binding strength with oxygen vacancy leads to more difficulties in completing the charge reaction. Therefore, in the case of zinc-ion battery cathode, the research findings stated that *δ*-MnO_2−*x*_-2.0 (the reduction time is 2 min) delivered the highest diffusion coefficient and the lowest reaction resistance, resulting in the most remarkable electrochemical performance (large specific capacity of 551.8 mA h g^−1^ at 0.5 A g^−1^, high-rate capability of 262.2 mA h g^−1^ at 10 A g^−1^) among all the samples, including *δ*-MnO_2_, *δ*-MnO_2−*x*_-0.5, *δ*-MnO_2−*x*_-2.0, *δ*-MnO_2−*x*_-5.0, as shown in Fig. 7c. Peng *et al.* regulated the Mn atomic coordination environment through electrochemical deposition and chemical reduction strategies.^98^ The optimized oxygen vacancy coordination environment can improve the reactivity, form a localized electric field, accelerate the ion/electron mobility, and realize an extremely high specific capacitance of 4.8 F cm^−2^/402.6 F g^−1^. It is expected that the coexistence of exfoliation and oxygen vacancies in *δ*-MnO_2_ would enlarge its electrochemical performance due to the large surface area and high electric/ion conductivity. Tang *et al.* proposed few-layer defective *δ*-MnO_2_ nanosheets (named *δ*-MnO_2_-CTAB) by using a redox reaction between cetyltrimethylammonium bromide (CTAB) and KMnO_4_.^99^ The exchange of CTA^+^ and K^+^ in the interlayer caused exfoliation of *δ*-MnO_2_ to obtain the ultrathin nanosheet structure (Fig. 7d). Meanwhile, the spatial steric effect of CTA^+^ weakened the van der Waals force between the layers, introducing many oxygen vacancies, which was confirmed by X-ray photoelectron spectroscopy (XPS) results. The synergistic effect of exfoliation and oxygen vacancies led to a larger specific surface area than pure *δ*-MnO_2_, providing more active sites, channels, and space for ion storage and transport. An ultra-high rate-capability of 77% was achieved as the current density was increased 50-fold (1 to 50 A g^−1^) (Fig. 7e). The intercalation of doping ions in *δ*-MnO_2_ will inevitably lead to the expansion or contraction of the lattice, resulting in lattice distortion and defects because of the different ionic radius or valence state, which is also called doping type vacancies. The synergy of ion pre-intercalation and oxygen vacancies in *δ*-MnO_2_ can also tremendously improve its electrochemical performance because of the enhanced structural stability and the fast charge transfer capability.^100,101^

Unlike the anion vacancy, the formation energy for cationic vacancy is higher, making its construction more difficult. The similarity is that the cationic vacancy in metal oxides can significantly regulate the electronic structure, increase the density of states near the Fermi level, and improve their electrical conductivity. Moreover, the relatively low atomic escape energy of ultra-thin two-dimensional materials makes it easier to construct cationic vacancies. Manceau *et al.* confirmed that Mn atoms migrate steadily from the interlayer position (above or below the Mn layer vacancy), and the remaining intralayer Mn atoms shift toward the vacancy at lower pH, leading to Mn vacancies in the nanosheets.^102^ On the basis of this research, Gao and coworkers investigated the amount of Mn vacancies by reassembling the as-exfoliated birnessite at different pH values.^103^ This observation in X-ray absorption near edge structure (XANES) spectra (Fig. 7f) confirmed the existence of mixed oxidation states of Mn^3+^/Mn^4+^ in the samples. The Mn surface Frenkel defect content was controlled to a value of 26.5% and 19.9% at pH = 2 and 4, respectively, which was in direct proportion to capacitance due to increase of the Mn^3+^/Mn^4+^ ratio and the reduced charge transfer resistance in 1 mol L^−1^ Na_2_SO_4_ electrolyte (Fig. 7g and h). Besides the defects mentioned above, many lattice defects can also significantly change the physical and chemical properties, such as lattice dislocation, expansion, and deformation. However, there is still a lot of room for exploration, and many difficulties need to be overcome.

### 3.4 Element doping

**Fig. 8:**
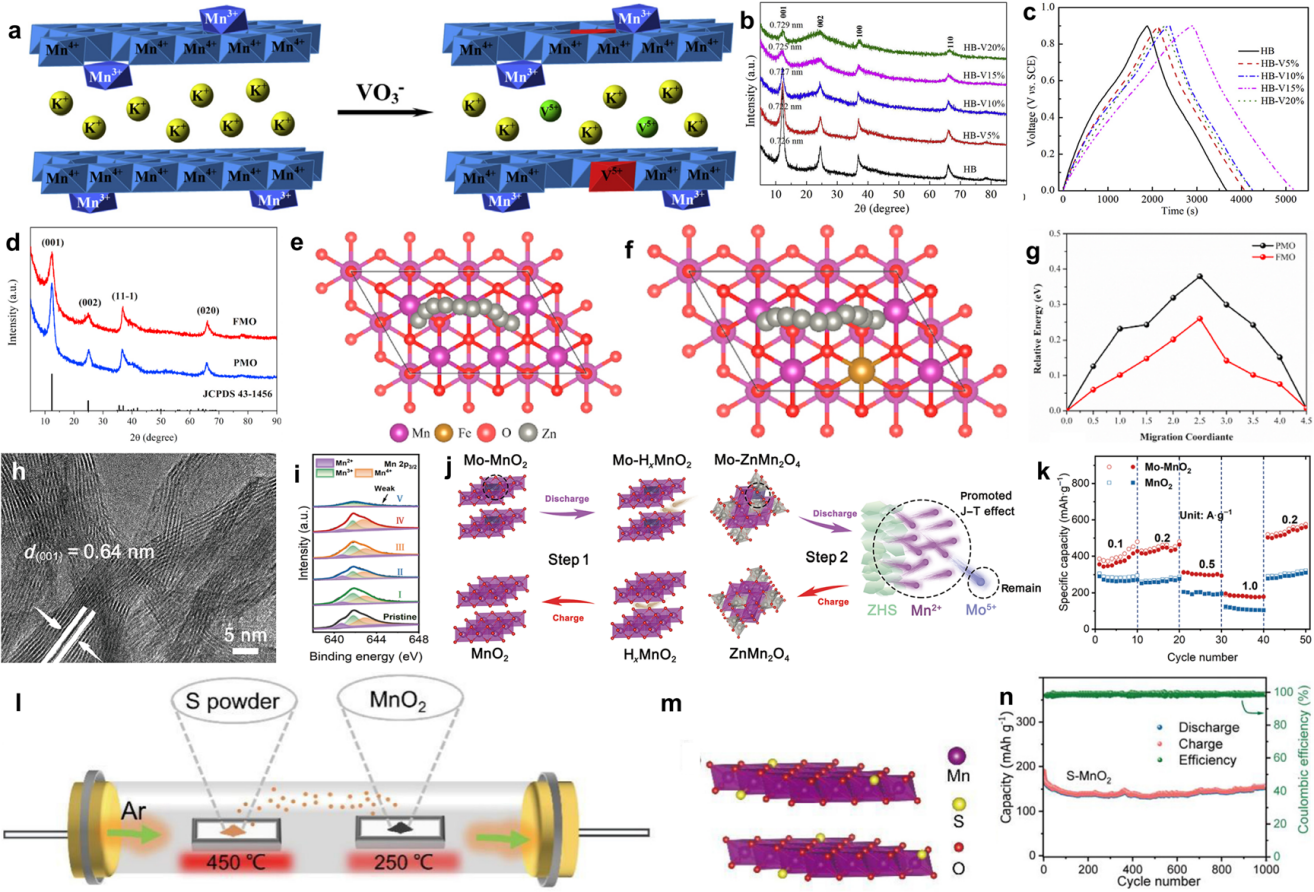
(a) Schematic diagram of V-doped birnessite. (b) XRD patterns of birnessites. (c) Charge/discharge profiles of HB, HB-V5%, HB-V10%, HB-V15%, and HB-V20% at 100 mA g^−1^.^109^ Reproduced with permission from ref. 109. Copyright 2015, Elsevier. (d) XRD spectra of PMO and FMO. (e and f) Zn ion diffusion path in PMO and FMO, respectively. (g) Lowest energy barrier for the diffusion of Zn ions through the FMO and PMO.^111^ Reproduced with permission from ref. 111. Copyright 2022, Elsevier. (h) TEM image of Mo–MnO_2_. (i) XPS of Mn atom. (j) Schematic of the mechanism for Zn//Mo–MnO_2_ during the charging/discharging process. (k) Rate test of MnO_2_ and Mo–MnO_2_.^112^ Reproduced with permission from ref. 112. Copyright 2022, Springer. (l) Illustration of the preparation process, (m) atomic structure model, and (n) cycling performance of S–MnO_2_.^115^ Reproduced with permission from ref. 115. Copyright 2022, Elsevier.

Recently, researchers attempted to improve the electrochemical performance of layer manganese dioxide by using element doping, such as V, Mo, Fe, Ag, Co, Ca, *etc.*^104–107^ Element doping can improve the conductivity of layer manganese oxide electrode material and enhance the electron transport ability inside the material. At the same time, doping can introduce additional redox reaction center sites to enhance the energy storage capacity of the material. In addition, element doping can also improve the interface reaction performance of the material, slow down the side reaction between the electrode material and the electrolyte, and improve the electrochemical stability.^108^ Liu and coworkers prepared V-doped birnessite with different contents of vanadium atoms and investigated the supercapacitance performance (Fig. 8a).^109^ With an increase of V/Mn molar, the crystallinity of birnessite decreased (Fig. 8b), and the relative proportion of Mn^3+^ first increased and then decreased, while the relative proportion of Mn^4+^ first decreased and then increased; that is, a high doping concentration will eventually lead to a decreased Mn^3+^/Mn^4+^ redox couple, which could result in a decrease in the specific capacitance. Therefore, the highest specific capacitance of 245 F g^−1^ was obtained for doped birnessite with a V/Mn molar ratio of 0.14 : 1 (HB-V15%), with excellent cyclic stability (Fig. 8c). Xia's group also investigated the electrochemical properties of Cr-doped *δ*-MnO_2_. The specific capacitance first increased and then decreased with a higher doping concentration.^110^ Li *et al.* proposed group VIII metal (Fe, Co and Ni) doped *δ*-MnO_2_ using the hydrothermal method for aqueous Zn-ion batteries.^111^ XRD results stated that Fe-doping (FMO) might introduce lattice disorders or defects (Fig. 8d), with no change in the nanoflower morphology. The zinc ion diffusion path in pure *δ*-MnO_2_ (PMO) and FMO was modeled (Fig. 8e and f), and the lower energy diffusion barrier of FMO compared with PMO is expected to result in faster diffusion kinetics (Fig. 8g). *In situ* XRD and *ex situ* TEM elucidated a highly reversible phase conversion (*δ*-MnO_2_ → Zn_*x*_MnO_2_ → ZnMn_2_O_4_/Mn_3_O_4_ → Zn_*x*_MnO_2_ → *δ*-MnO_2_), giving rise to a high specific capacity of 338.2 mA h g^−1^ at 1 A g^−1^ with a capacity retention of 86.3% after 200 cycles, like that of Co and Ni-doping MnO_2_ electrode. However, the capacity of *δ*-MnO_2_ is mainly limited by the one-electron reaction of Mn^4+^/Mn^3+^ redox, which could be boosted through reduction of Mn oxygen state. Therefore, Xia *et al.* reported MnO_2_ based cathode with two-electron redox reaction through Mo doped *δ*-MnO_2_ (Mo–MnO_2_).^112^ Mo doping had no effect on the crystal structure and interlayer spacing (Fig. 8h), illustrating that the Mo atom partially replaced the Mn atom in the lamellar. The XPS result stated the existence of mixed valence states of Mn^4+^, Mn^3+^ and Mn^2+^ due to the introduction of Mo^5+^ dopant (Fig. 8i). Density functional theory (DFT) calculations stated that the narrowed band gap created excellent electric conductivity. The zinc ion storage mechanism of Mo–MnO_2_ (Fig. 8j) was based on a reversible two-step two-electron redox deduced by ex-site XRD, XPS and SEM images. As the cathode of the zinc-ion battery, the capacity of 652 mA h g^−1^ at 0.2 A g^−1^ and rate capability was better than that of pure MnO_2_ (Fig. 8k). Additionally, Ye *et al.* reported Se-dopant layer manganese dioxide (Se–MnO_2_) as a Zn-ion battery. The doping content of Se can control the ratio of H^+^ intercalation in MnO_2_ and inhibit the formation of ZnMn_2_O_4_ by-products, further creating excellent long-term cycle stability.^113^ Notably, element doping can increase the specific capacity and improve the stability, mainly because it greatly inhibits the elongations of Mn–O bonds along the *z*-axis to reduce Jahn–Teller distortion. Recently, Yang's group confirmed the role of Mg substitution in layered K_0.5_MnO_2_. Mg^2+^ substitution could promote the formation of Mn^3+^ ions with electron configurations eg^(1)^t_2_g^(3)^ and t_2_g^(4)^, effectively alleviating the structural deformation usually caused by Jahn–Teller distortion.^114^ Beyond that, Zhao *et al.* doped sulfur (S) anion and introduced oxygen vacancies into the layered manganese dioxide lattice by defect engineering. The preparation process and atomic structure model of S–MnO_2_ are shown Fig. 8l and m, respectively.^115^ S doping increased the intrinsic electron conductivity, and enhanced the diffusion kinetics of Zn ions, achieving good rate capability and excellent endurance cycle capacity of over 1000 cycles at a current density of 3 A g^−1^ (Fig. 8n). Nevertheless, the doping process may introduce new impurities, which may affect the purity and stability of the material, and doped elements may migrate or fall off at high temperature or humidity. Therefore, the doping conditions and element types need to be further studied.

### 3.5 Expanding interlayer spacing

The capacitance of layer manganese dioxide acting as the electrode material of the supercapacitor is mainly composed of surface EDL capacitance, surface redox pseudocapacitance and intercalated pseudocapacitance in the bulk. Meanwhile, the capacity is dominated by the (de)intercalation of charge carriers as the electrode material of battery. Therefore, a reasonable interlayer distance is one of the most indispensable parameters to obtain high electrochemical performance. A comprehensive review of the literature stated that the pre-intercalation strategy involving the insertion of water molecules,^116^ guest ions or polymers into the layer manganese dioxide host structure before cycling is an advanced way to boost interlayer distance.^117–119^ Specially, Zhao *et al.* reviewed the interlayer pre-intercalation strategy and results in manganese oxides.^120^ The interlayer pre-intercalation can simultaneously improve the electronic conductivity, activate more active sites, promote diffusion kinetics, stabilize structure integrity, and depress the phase transition of MnO_2_ cathode materials.

#### 3.5.1 Guest ion pre-intercalation

**Fig. 9:**
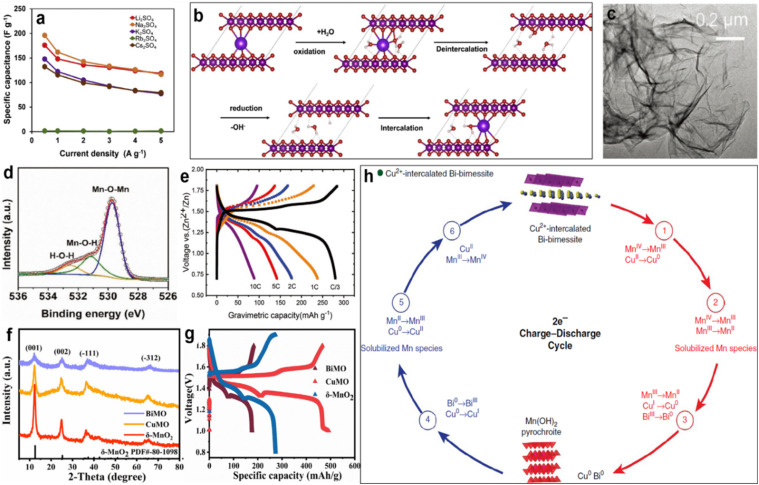
(a) Specific capacitance as a function of current densities for various electrolytes. (b) Schematic diagram of the layer manganese oxide charge storage mechanism *via* electrochemical intercalation/deintercalation of alkali cation.^124^ Reproduced with permission from ref. 124. Copyright 2020, Elsevier. (c) Low resolution TEM image of K_0.27_MnO_2_·0.54H_2_O. (d) XPS spectrum of O 1s. (e) Discharge–charge curves at various current rates.^125^ Reproduced with permission from ref. 125. Copyright 2021, Wiley-VCH. (f) XRD pattern of as-synthesized CuMO, BiMO, and *δ*-MnO_2_. (g) Charge–discharge curves of CuMO, BiMO, and *δ*-MnO_2_ at 0.1 A g^−1^ (in the second cycle).^128^ Reproduced with permission from ref. 128. Copyright 2022, Elsevier. (h) Electrochemical reactions for the regeneration cycle of Cu^2+^-intercalated Bi-birnessite.^129^ Reproduced with permission from ref. 129. Copyright 2017, Springer Nature.

Intercalated guest ions including single valent (Li^+^, Na^+^, and K^+^) and multivalent metal ions (Ca^2+^, Zn^2+^, La^3+^, and Ce^3+^) could reduce the bandgap between the valence and conductive bonds, contributing to higher intrinsic electronic/ionic conductivity. Beyond that, the functionality of pre-intercalation has been proposed, such as creating active sites, facilitating ionic diffusion kinetics and stabilizing the crystal structure during the charge/discharge process.^121–123^ Ma *et al.* synthesized Li^+^-intercalated *vis* a one-pot process and studied the electrochemical behavior in the sulfate-based electrolyte (Li_2_SO_4_, Na_2_SO_4_, K_2_SO_4_, Rb_2_SO_4_, and Cs_2_SO_4_).^124^ Li-manganese dioxide displayed the highest charge storage capacity in 0.5 mol L^−1^ Na_2_SO_4_, which mainly depended on the synergistic effect of the hydrated ion diameter of the electrolytic cation, the active site utilization of Mn and the change in the oxidation state (Fig. 9a). The charge storage mechanism of the layered manganese dioxide nanosheet is shown in Fig. 9b. Additionally, single valent alkali ions pre-intercalated layered MnO_2_ was prepared to use as a cathode in zinc ion batteries. Potassium ion pre-intercalated layered MnO_2_ nanosheet (K_0.27_MnO_2_·0.54H_2_O, known as KMO) was prepared by Simon's group *via* a rapid molten salt method (Fig. 9c).^125^ The XPS spectrum illustrated that the interlayer water molecules formed chemical bonds with the MnO_2_ layers (Fig. 9d). The pillaring effect of K^+^ and water crystals benefited for the good structure stability of KMO and a rapid diffusion of cations in the KMO structure, contributing to high power capability (90 mA h g^−1^ at 10C) and good cycling stability (91% after 1000 cycles), as shown in Fig. 9e. However, the univalent ion pre-intercalation has a limitation on the expansion of layer spacing. Multivalent metal ions (*e.g*., Co^2+^, Pb^2+^, and Y^3+^) intercalated layer manganese dioxides improve the electrochemical characteristics.^14,126,127^ However, it is a pity that these intercalants are toxic and/or expensive. Therefore, Long *et al.* produced multivalent metal ions (Cu^2+^ and Bi^3+^) intercalated manganese dioxide (CuMO and BiMO) using the hydrothermal method for aqueous zinc ion batteries.^128^ The XRD pattern indicated the co-intercalation of copper ion, bismuth ion and water molecules (Fig. 9f). CuMO displayed the highest capacity of 493.3 mA h g^−1^ at 100 mA g^−1^ because of the additional capacity contribution based on the transformation reaction between CuMO and metallic Cu. BiMO delivered the longest cycle life with a capacity retention of 98.6% after 1100 cycles at 1 A g^−1^ due to the pillar role of pre-intercalated Bi^3+^ and water molecules, which were superior to that of pure *δ*-MnO_2_ (Fig. 9g). Moreover, lower overpotential of CuMO and BiMO manifested faster reaction kinetics compared with *δ*-MnO_2_. Yadav *et al.* reported Cu^2+^ intercalated Bi-birnessite (Bi–*δ*-MnO_2_) cathode using metallic Cu as an additive for the zinc battery to integrate the excellent electrochemical performances of the electrode material.^129^ During charging, Cu transferred to Cu^2+^ to intercalate in the interlayer regions of Bi-*δ*-MnO_2_ at the redox potential, and was reduced to Cu^0^ with the Mn(OH)_2_-layered material during discharge (Fig. 9h). Cu addition contributed towards the stability and electronic/ionic conductivity of Bi-*δ*-MnO_2_, resulting in an excellent areal capacity and cycle life. This wonderful idea can be applied to layered materials in other fields. Although the intercalation of metal ion in layered MnO_2_ can effectively enhance the stability and electronic/ionic conductivity, the expanded layer spacing is still less than 0.8 nm, which seriously prevents the movement of large carriers, such as Ca^2+^, Mg^2+^ and Al^3+^. It is particularly important to further expand the layer spacing and maintain stability.

#### 3.5.2 Organic ions/polymer pre-intercalation

**Fig. 10:**
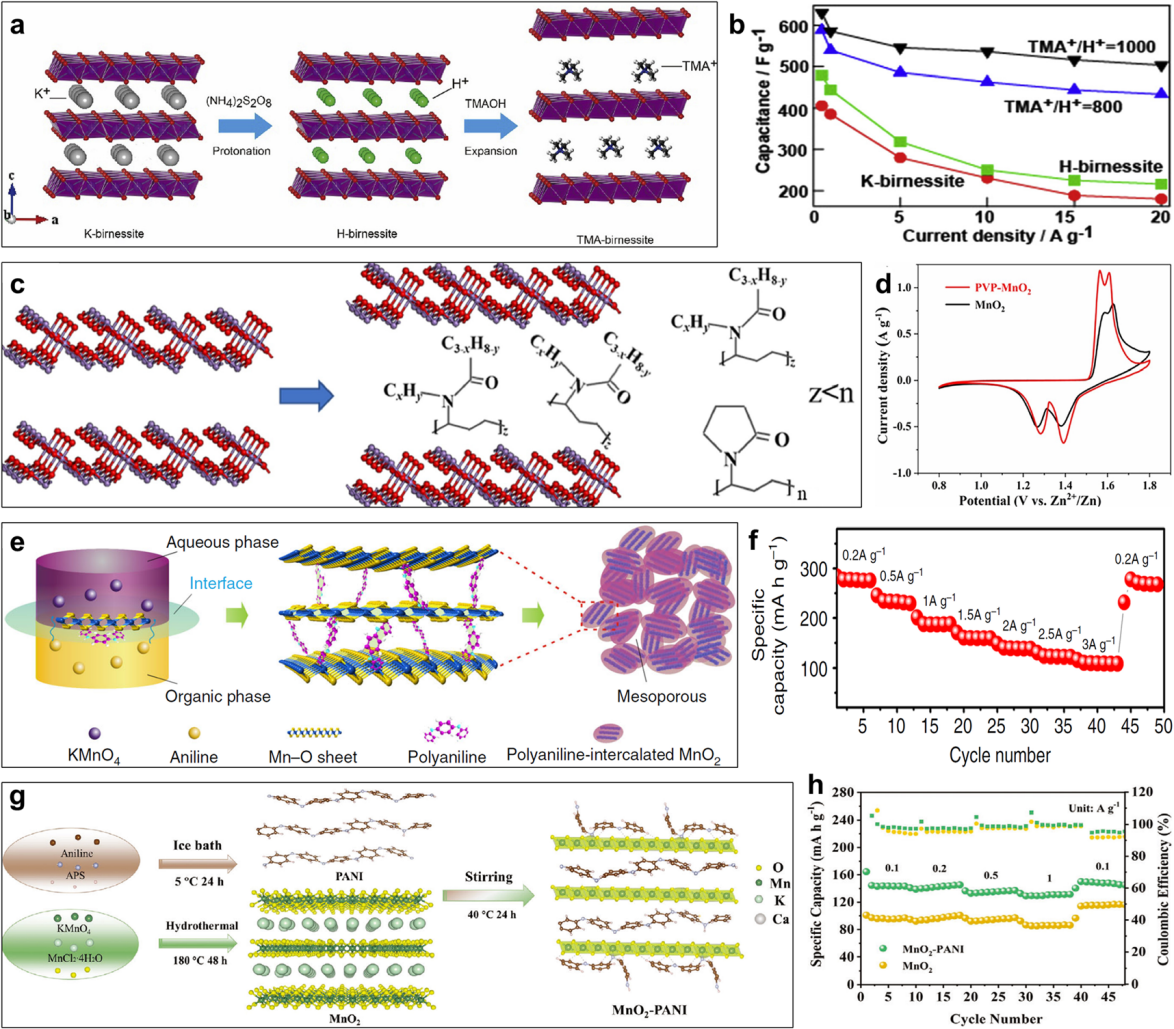
(a) Schematic illustration of the ion-exchange process. (b) Comparison of specific capacitance at different current densities.^131^ Reproduced with permission from ref. 131. Copyright 2017, Elsevier. (c) Schematic of PVP intercalation in *δ*-MnO_2_. (d) CV profiles of MnO_2_ and PVP-MnO_2_.^133^ Reproduced with permission from ref. 133. Copyright 2023, Wiley-VCH. (e) Schematic of the expanded intercalated structure of PANI-intercalated MnO_2_ nanolayers. (f) Rate performance of PANI-intercalated MnO_2_.^136^ Reproduced with permission from ref. 136. Copyright 2018, Springer Nature. (g) Schematic of the synthesis of MnO_2_–P organic–inorganic nanosheets. (h) Rate performance of MnO_2_ and MnO_2_–P.^137^ Reproduced with permission from ref. 137. Copyright 2023, Wiley-VCH.

The larger size of organic ions or polymer compared with metal ions can effectively extend the layer spacing of layered manganese dioxide to 0.9 nm, and even 1.3 nm. In general, organic ions/polymers are difficult to intercalate in layered manganese dioxide by the one-step hydrothermal method and calcination method because they tend to form composites rather than intercalation. Therefore, ion-exchange and interface reactions are the most commonly used methods. Based on the swelling and delamination behaviors of the protonated birnessite in tetrabutylammonium hydroxide (TMAOH) solutions, TMA^+^ intercalated birnessite (TMA-birnessite) with large layer spacing was prepared *via* ion-exchange reactions for electrochemical energy storage.^33,43,130^ Zhao *et al.* also used the ion exchange method to prepare TMA-birnessite with different molar ratio of TMA^+^/H^+^ for pseudocapacitors (Fig. 10a).^131^ When TMA^+^/H^+^ increased up to 1000, H^+^ is almost totally replaced by TMA^+^, resulting in an enlarged interlayer spacing of 0.96 nm. The ion diffusion tunnels were enlarged thanks to the expansion of interlayer spacing, and the layer interaction and resistance of charge diffusion decreased. Therefore, TMA^+^/H^+^ = 1000 displayed the largest specific capacitance of 585 F g^−1^ and the best rate capability of 86% from 1 to 20 A g^−1^ (Fig. 10b). Wang's group reported TMA-birnessite through a two-step ion-exchange reaction from K-birnessite to H-birnessite to TMA-birnessite for bivalent Mg-ion storage.^132^ The exchange of K^+^ and TMA^+^ in the interlayer made the interlayer spacing expand from 0.70 to 0.97 nm, which was beneficial to a faster diffusion of Mg^2+^ and lower overpotential. TMA-birnessite delivered twice as much specific capacity as K-birnessite with excellent rate performance. Polymer-intercalated layered manganese dioxide have been intensively investigated to further enhance layered spacing. Zhang and co-workers proposed polyvinylpyrrolidone (PVP) pre-intercalation *δ*-MnO_2_ (PVP–MnO_2_) for Zn-ion batteries (Fig. 10c).^133^ The as-prepared PVP-MnO_2_ exhibited a terrace-shape hybrid superlattice structure and an expanded interlayer spacing of 1.14 nm more than TMA-birnessite reported previously, which was beneficial to Zn^2+^/H^+^ co-insertion. Obviously pronounced redox intensity and reduced potential hysteresis in CV profiles (Fig. 10d) illustrated the improved electrochemical reactivity and diffusion kinetics due to PVP pre-intercalation. PVP-MnO_2_ delivered impressive electrochemical performance, such as a high specific capacity (317.2 mA h g^−1^ at 0.125 A g^−1^), improved rate performance (106.1 mA h g^−1^ at 12.5 A g^−1^) and excellent cycle stability (capacity retention of almost 100% after 20 000 cycles at 10 A g^−1^). Polyquaternium-2 (PQN2) was intercalated in layered manganese oxide through a delamination/reassembling process, resulting in an expanded interlayer spacing of 0.94 nm.^134^ Beyond that, Xia's group designed polyaniline-intercalated layered manganese dioxide using a simple one-step inorganic/organic interface reaction.^135,136^ The formation of PANI restricted the growth of 2D MnO_2_, eventually resulting in a mesoporous structure (Fig. 10e).^136^ A high specific capacity of 298 mA h g^−1^ was obtained after subsequent cycles thanks to the typical nanosize (∼10 nm), expanded interlayer space (∼1.0 nm), uniform mesostructure (∼4 nm) and polymer-reinforced layered structure, which was barely the theoretical specific capacity of 308 mA h g^−1^. Meanwhile, an amazing rate performance (Fig. 10f) and cycle stability was observed without phase transformation and collapse of the layered structure. However, the yield of the interface reaction is low, and polyaniline intercalation and coating coexist. This causes difficulties to quantify the insertion amount of polyaniline. Zuo *et al.* developed MnO_2_-PANI hybrid cathode (MnO_2_–P) with PANI intercalation and coating for Ca-ion batteries.^137^ The presence of PANI improves the conductivity and enlarges the interlayer spacing, facilitating electron transport and Ca^2+^ diffusion. Moreover, the formed Mn–N bond based on the PANI coating can enhance structural stability (Fig. 10g). The above characteristics gave excellent electrochemical performances to the MnO_2_-PANI hybrid cathode, including high capacity, outstanding rate performance (Fig. 10h) and a long-term cycle. These results indicate that the interlayer spacing is efficiently expanded in favour of ion transport by the organic ions/polymer pre-intercalation strategy. However, the current research is limited to expanding the layer spacing, and the correlation between layer spacing and electrochemical properties of aluminum has not been studied. In addition, there are still some questions that need to be further clarified, such as whether the intercalation amount has a negative effect on the ion transport rate and mechanical structure stability, and how to achieve accurate regulation of the intercalation amount.

## 4. Summary and outlook

Layer manganese dioxide is still one of the preferred materials for supercapacitors and various ion batteries. Researchers have proposed a variety of strategies on the macro and micro levels to improve the electrochemical performance. In this review, we summarized the latest advances in the study of charge-storage mechanisms and related improvement strategies, including preparing conductive composite, novel nano-structural design, defect engineering, element doping and expanding interlayer spacing. These strategies improve electrical/ionic conductivity, increase electrochemically active sites and stabilize the layer structure, while providing the foundation and inspiration for future research. However, there is a long way to go for attaining the theoretical electrochemical performance levels for the available practical electrodes, for which advanced strategies should be employed.

### 4.1 Porous layer-by-layer assembly

The low utilization of layer manganese dioxide is a challenge to obtain high capacitance/capacity. However, it is feasible to maximize the specific surface area by assembling the manganese dioxide layer with other conductive two-dimensional material to enhance the utilization of layer manganese dioxide as an electrode. Furthermore, creating pores on the layers can achieve a desirable ionic conductivity. This idea deserves more experiments.

### 4.2 Special element doping

It has been confirmed that element doping (metal or non-metal elements) is an effective way to improve the performance of layer manganese dioxide. It is challenging to uncover the relationship between doping concentration and electrochemical properties. In addition, element doping technology can be combined with other modification strategies to further improve the performance of manganese oxide electrode materials, such as nanostructure design, layer engineering, *etc.*

### 4.3 Coating and pre-intercalation integrated design

The pre-intercalation of polymer in layer manganese dioxide can expand the layer spacing to about 1.0 nm, facilitate the fast transfer of electrolyte ion, and accelerate the kinetic process. It will be interesting to use conductive polymer to pre-intercalate manganese dioxide and to achieve coating at the same time, which may facilitate the simultaneous transfer of electrons and ions. The interaction between layer spacing and electrochemical properties needs further investigation.

### 4.4 Research on energy storage mechanism

Layer manganese dioxide exhibits different energy storage mechanism as an electrode material of various kinds of batteries, which urgently needs to be understood and explored using various spectral approaches. Meanwhile, the energy storage mechanism of manganese dioxide may be different within different potential windows, which can be studied according to the high voltage characteristics of high concentration aqueous electrolytes.

## Data availability

The data that support the findings of this study are available from the corresponding author upon reasonable request.

## Author contributions

Y. Z., H. X., X. H. Z., Z. G. and B. B. X. developed the concept and performed literature scoping and review with M. E., X. Z. The copyright of literatures were acquired by H. X., X. Z. and K. J. Figures were processed by Y. Z., X. Z. and K. J. All authors were involved in drafting the manuscript with the special assistances from M. E. and K. J. to adjust the format of manuscripts. X. H. Z., Z. G. and B. B. X. critically reviewed the context. The manuscript was proofread by M. E., X. H. Z. and Z. G. Y. Z., Z. G. and B. B. X. provided the funding, administration and supervision to support the project. The authors extend their appreciation to the Deanship of Scientific Research at Northern Border University, Arar, KSA for funding this research work through the project number “NBU-FFR-2025-80-01”.

## Conflicts of interest

There are no conflicts to declare.
